# Ageing, metabolism and the intestine

**DOI:** 10.15252/embr.202050047

**Published:** 2020-06-21

**Authors:** Maja C Funk, Jun Zhou, Michael Boutros

**Affiliations:** ^1^ Division Signaling and Functional Genomics German Cancer Research Center (DKFZ) Heidelberg University Heidelberg Germany

**Keywords:** ageing, epithelial barriers, intestinal homeostasis, metabolism, stem cells, Metabolism, Molecular Biology of Disease, Regenerative Medicine

## Abstract

The intestinal epithelium serves as a dynamic barrier to the environment and integrates a variety of signals, including those from metabolites, commensal microbiota, immune responses and stressors upon ageing. The intestine is constantly challenged and requires a high renewal rate to replace damaged cells in order to maintain its barrier function. Essential for its renewal capacity are intestinal stem cells, which constantly give rise to progenitor cells that differentiate into the multiple cell types present in the epithelium. Here, we review the current state of research of how metabolism and ageing control intestinal stem cell function and epithelial homeostasis. We focus on recent insights gained from model organisms that indicate how changes in metabolic signalling during ageing are a major driver for the loss of stem cell plasticity and epithelial homeostasis, ultimately affecting the resilience of an organism and limiting its lifespan. We compare findings made in mouse and *Drosophila* and discuss differences and commonalities in the underlying signalling pathways and mechanisms in the context of ageing.

## Graphical abstract

The intestinal epithelium plays important roles in nutrition, signaling and as a barrier to the environment. Intestinal stem cells are crucial to replace epithelial cells and to maintain epithelial homeostasis. This review discusses how aging and metabolism affect stem cell function and epithelial homeostasis in the mouse and fly intestine.

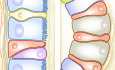

## Introduction

**Figure 1:**
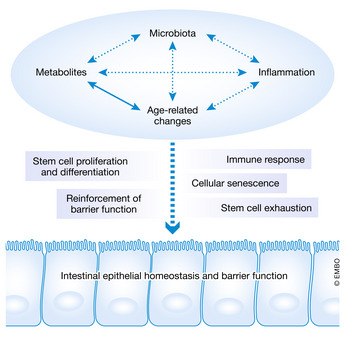
The gut functions as a dynamic barrier to the environment with signal integration properties The gut functions as a chemical and physical barrier to the environment and serves as an integration site to respond to diverse intrinsic as well as extrinsic stimuli. These stimuli display a wide range of metabolites, age‐related changes, microbiota as well as inflammation‐associated processes. The resulting functional consequences include adaption in stem cell and proliferation, immune responses, reinforcement of the barrier function, cellular senescence as well as stem cell exhaustion. Functional consequences constantly affect the epithelial homeostasis and subsequently the barrier function of the intestine, eventually leading to epithelial barrier defects.

The intestine is one of the most versatile epithelia in our body; it is involved in many physiological processes, including nutrient uptake as well as immune modulation and displays the interface to a complex commensal microbiome. Importantly, the intestine is also responsible for the absorption of nutrients throughout our lifetime. It serves as an important physical and chemical barrier to the environment, while also functioning as an integration site that responds to diverse physiological and pathophysiological stimuli. The stimuli include a wide range of metabolites, signals from the immune system and communication with the resident bacteria (Fig 1). Together, the crosstalk between these stimuli can dynamically impact the intestine in response to environmental or organismal challenges. Ageing poses an additional challenge for a constantly renewing epithelium and its protective function. The resulting functional consequences include adaptation of stem cell (SC) proliferation and differentiation, SC exhaustion, cellular senescence, immune and inflammatory responses as well as the reinforcement of epithelial barrier function (Fig 1). Importantly, these factors influence the epithelial homeostasis and barrier function of the intestine, and their aberrant regulation can lead to epithelial barrier defects and subsequent detrimental systemic effects on the entire organism (Nicoletti, 2015). Thus, identifying the mechanisms leading to robust epithelial function is of great importance and biomedical interest to counteract increased susceptibility to gastrointestinal disorders with age (Sovran *et* *al,* 2019) and to treat patients with metabolic syndromes (Thaiss *et* *al,* 2018).

The fruit fly *Drosophila melanogaster* and mice are widely used genetic model systems to study human diseases (Aitman *et* *al,* 2011; Lehner, 2013). Over the last decades, *Drosophila* and mice have contributed important insights into diverse biological processes in the intestine. This review focuses on intestinal homeostasis, metabolism and ageing, highlighting both similarities and differences between vertebrates and invertebrates. In addition, we discuss the potential consequences of these interactions on the epithelial barrier and, thus, organismal effects.

## Principal concepts of intestinal homeostasis, metabolism and ageing

### Intestinal homeostasis

**Figure 2:**
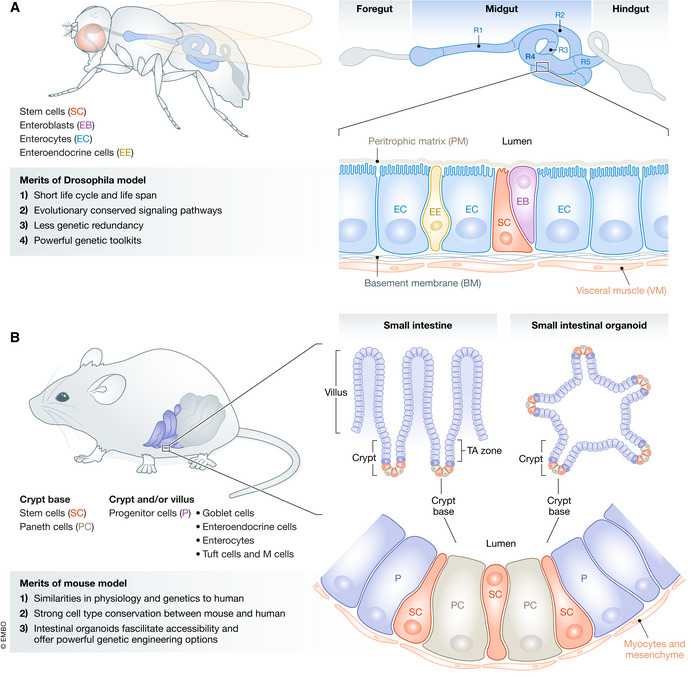
Schematic representation of the anatomy of *Drosophila* and mouse intestine (A) The Drosophila digestive tract is composed of foregut, midgut and hindgut. The cell types in the adult intestine include: stem cells (SC), enteroblasts (EB), enterocytes (EC) and enteroendocrine cells (EE). The intestinal epithelium is surrounded by visceral muscles and peritrophic membrane that separates the intestinal cells from bacteria presented in the lumen. (B) The epithelium of the mouse small intestine is structured into the crypt region, the transit amplifying (TA) region and the villus region. Stem cells (SC) of the intestine are located in the crypts and are surrounded by Paneth cells (PC), which provide essential growth factors to the SC and are part of the stem cell niche. Transit amplifying cells that have left the crypt region are pushed upwards to the villi and driven towards differentiation in the different cell types of the intestinal epithelium, including goblet cells (GC), enteroendocrine cells (EE), tuft cells, M cells and enterocytes (EC). The intestinal epithelium is underlined by a muscle layer and mesenchyme.

Epithelial homeostasis is dependent on a balance between intestinal stem cell (ISC) self‐renewal, progenitor differentiation, cell shedding and apoptosis (see Fig 2 for a schematic of fly and mouse intestine). In this context, the capacity of ISCs to decide between self‐renewal and differentiation allows for dynamic response and remodelling of the epithelium in response to external stimuli. Both, the *Drosophila* and mouse intestine, undergo rapid cell turnover, with a self‐renewal rate of 3–5 days in the murine intestine (Cheng & Leblond, 1974). In the murine intestine, the main driver for this high proliferation is Wnt ligands, mainly secreted by Paneth cells (PCs) and the underlying mesenchyme, with both Wnt sources seemingly functionally redundant for the maintenance of intestinal homeostasis (Sato *et* *al,* 2011; Kabiri *et* *al,* 2014; Valenta *et* *al,* 2016). In *Drosophila*, the visceral muscles similarly produce the Wnt ligand wingless (wg), which has also been implicated in ISC maintenance (Lin *et* *al,* 2008). In *Drosophila,* as well as in the mouse intestine, tissue homeostasis is based on a neutral competition between symmetrically dividing SCs (Snippert *et* *al,* 2010; de Navascués *et* *al,* 2012). In differentiating progenitors, Notch signalling is responsible for suppressing the secretory lineage and promoting enterocyte (EC) differentiation in both, the *Drosophila* and mouse systems (Milano *et* *al,* 2004; Fre *et* *al,* 2005; Guo & Ohlstein, 2015). Upon damage, or in response to external cues, the equilibrium is shifted towards self‐renewal or proliferation, as will be discussed later. In summary, homeostasis of the intestinal epithelium is key for its functionality and maintenance of the epithelial barrier, which is essential for organismal health.

### Intestinal metabolism

Caloric restriction (CR) has been proposed to promote longevity in a wide range of organisms (Fontana & Partridge, 2015), and current efforts aim to shed light on the molecular mechanisms underlying this organismal effect. The intestinal epithelium is in direct contact with nutrients and metabolites, representing a first site where CR, or other diet regimes, could impact on the organism. In recent years, new insights have been gained into how different nutritional states can influence ISC function and thereby epithelial homeostasis. Two different modes of response can be distinguished: a direct influence of metabolites on ISC function by modulating signalling pathways and an indirect response of ISCs on changes in the dietary status to remodel the cellular composition of the epithelium. Moreover, the response can be ISC‐intrinsic or mediated via other epithelial cell types, such as neighbouring Paneth cells in mice or ECs in *Drosophila*.

### Intestinal ageing

In a rapidly renewing tissue, ISCs are particularly challenged to keep up with the speed of proliferation for self‐renewal and progenitor differentiation (Cheng & Leblond, 1974). Additionally, over the lifespan of the organism, ISCs suffer from increased replicative age, including the accumulation of cellular and DNA damage that can occur during every round of replication (Liu & Rando, 2011). Thus, ISCs are highly prone to stem cell exhaustion, an integrative hallmark of ageing (López‐Otín *et* *al,* 2013). This ageing phenotype is characterized by a reduced self‐renewal capacity of ISCs, a diminished tissue self‐repair function after damage and misbalanced progenitor differentiation. These consequences influence the epithelial homeostasis and barrier function, which is frequently observed in the elderly as leaky gut syndrome (Kiefer & Ali‐Akbarian, 2004). In addition to cell‐dependent stem cell exhaustion, metabolism can also impact on intestinal homeostasis. Since the basal metabolic rate and nutrient usage change during organismal ageing (Henry, 2000; Houtkooper *et* *al,* 2011), metabolism might have additional effects on the ageing tissue, causing changes in ISC function and progenitor differentiation, as well as on how cells respond to external and internal stimuli.

*Drosophila* and mouse, two widely used genetic model systems, show common and distinct features that are essential for intestinal homeostasis, providing a ground for cross‐species investigation to unravel evolutionarily conserved mechanisms and the fundamental concepts of intestinal homeostasis. Nearly all genes mentioned in this review have homologs in the human genome (Tables 1 and 2), indicating that conserved mechanisms between mouse and fly are likewise relevant for human intestinal homeostasis and ageing.

**Table 1:** *Drosophila* genes discussed in the review with their predicted homolog in mouse and human (Homology score based on flybase.org algorithm)

*Drosophila* gene	Mouse homolog	Homology score	Human homolog	Homology score
Metabolism				
Ahcy	Gm4737	+++	AHCY	+++
AhcyL1	AhcyL1	+++	AHCYL1	+++
AhcyL2	AhcyL2	+++	AHCYL2	+++
Crtc	Crtc1	+++	CRTC1	+++
mGluR	Grm3	+++	GRM3	+++
CanB	Ppp3r1	+++	PPP3R1	+++
Drp1	Dmn1 l	+++	DNM1L	+++
Gcn2	Eif2ak4	+++	EIF2AK4	+++
park	Prkn	+++	PRKN	+++
Pgam5	Pgam5	+++	PGAM5	+++
Pgant4	Galnt10	+++	GALNT10	+++
Pink1	Pink1	+++	PINK1	+++
Signalling				
AMPKα	Prkaa2	+++	PRKAA2	+++
Ilp3	Igf1	+	INS	+
InR	Insr	+++	INSR	+++
Pi3K21B	Pik3r3	+++	PIK3R3	+++
srl	Pprc1	+++	PPRC1	+++
Tor	Mtor	+++	MTOR	+++
Thor	Eif4ebp1	+++	EIF4EBP1	+++
Dl	Dll1	+++	DLL1	+++
N	Notch1	+++	NOTCH1	+++
msn	Tnik	+++	TNIK	+++
wts	Lats2	+++	LATS1	+++
yki	Yap1	+++	YAP1	+++
Upd3	Il‐6	N/A	IL‐6	N/A
hop	Jak2	+++	JAK2	+++
Stat92E	Stat5b	+++	STAT5B	+++
bsk/JNK	Mapk8	+++	MAPK10	+++
Myc	Myc	++	MYC	++
Other functions				
Brf	Brf1	+++	BRF1	+++
eEF2	Eef2	+++	EEF2	+++
FoxK	Foxk1	+++	FOXK2	+++
Ire1	Ern1	+++	ERN1	+++
Xbp1	Xbp1	+++	XBP1	+++
Atg1	Ulk1	+++	ULK1	+++
bbg	Pdzd2	++	PDZD2	+
Col4a1	Col4a1	+++	COL4A6	+++
crc	ATF4	++	ATF4	++
Piwi	Piwil1	+++	PIWIL3	+++
prom	Prom1	+++	PROM2	+++
Tk	Tac1	+	TAC1	+
Tg	Tgm1	+++	TGM1	+++
Wdr62	Wdr62	+++	WDR62	+++

The table is also included in the Appendix, where all genes are linked to their respective NCBI gene entry. Homology score: 1–2: +, 3–5: ++, 6–15: +++.

**Table 2:** Mouse genes discussed in the review with their predicted homolog in *Drosophila* and human (Homology score based on flybase.org algorithm)

Mouse gene	*Drosophila* homolog	Homology score	Human homolog	Homology score
Metabolism				
Stk11 (Lkb1)	Lkb1	+++	STK11	+++
Mpc1	Mpc1	+++	MPC1	+++
Pdk4	Pdk	+++	PDK4	+++
Cpt1a	whd	+++	CPT1A	+++
Ppard	Eip75B	++	PPARD	+++
Ppara	Eip75B	++	PPARA	+++
Sirt1	Sirt1	+++	SIRT1	+++
Hmgcs2	HMGS	+++	HMGCS2	+++
Signalling				
Wnt3	wg	+	WNT3	+++
Lgr5	rk	+++	LGR5	+++
Ctnnb1	arm	+++	CTNNB1	+++
Notum	Notum	+++	NOTUM	+++
Notch1	N	+++	NOTCH1	+++
Atoh1	cato	+++	ATOH1	+++
Sox9	Sox100B	++	SOX9	+++
Mapk14 (p38)	p38b	+++	MAPK14	+++
Map2k6 (MKK6)	lic	+++	MAP2K6	+++
Trp53	p53	++	TP53	+++

The table is also included in the Appendix, where all genes are linked to their respective NCBI gene entry. Homology score: 1–2: +, 3–5: ++, 6–15: +++.

## Intestinal physiology in *Drosophila* and mouse

The mammalian and *Drosophila* intestines share fundamental similarities, such as food digestion, absorption, immune defence and host–microbe symbiosis (Marianes & Spradling, 2013; Dutta *et* *al,* 2015). Additionally, the intestine of *Drosophila* and mice houses stem cells that utilize similar mechanisms to ensure homeostatic renewal of tissue and injury‐induced regenerative responses. These mechanisms include the activity of highly conserved signalling pathways in the regulation of stem cell functions. For example, Notch controls ISC maintenance and EC differentiation (Fre *et* *al,* 2005; Stanger *et* *al,* 2005; Guo & Ohlstein, 2015; Beumer & Clevers, 2016), epidermal growth factor (EGF) signalling activates ISCs to proliferate (Buchon *et* *al,* 2010; Biteau & Jasper, 2011; Jiang *et* *al,* 2011; Jiang & Edgar, 2012; Jin *et* *al,* 2015; Smith *et* *al,* 2016), Wnt/wingless signalling governs ISC maintenance as well as proliferation (Lin *et* *al,* 2008; de Lau *et* *al,* 2011; Cordero *et* *al,* 2012; Clevers, 2013; Guo *et* *al,* 2016), and BMP/Dpp signalling restricts ISC proliferation and promotes cell differentiation (Haramis *et* *al,* 2004; He *et* *al,* 2004; Guo *et* *al,* 2013; Zhou *et* *al,* 2015; Ma *et* *al,* 2019). In *Drosophila*, ISC proliferation and differentiation is additionally regulated by Unpaired/JAK/STAT signalling (Buchon *et* *al,* 2009; Jiang *et* *al,* 2009; Cordero *et* *al,* 2012; Zhai *et* *al,* 2017). The *Drosophila* intestinal epithelium is devoid of mesenchyme and several specialized cell types that are present in the mouse intestine, such as Paneth cells, goblet cells (GCs) and Tuft cells (Fig 2) (Zwick *et* *al,* 2018). While murine intestinal stem cells rely on niche factors (Wnt, Notch, EGF) provided by the mesenchyme and Paneth cells, the *Drosophila* intestine relies on visceral muscles (VMs) and enteroblasts (EBs) to produce these niche factors for ISC proliferation and differentiation to maintain epithelial homeostasis (Lin *et* *al,* 2008; de Lau *et* *al,* 2011; Cordero *et* *al,* 2012; Zhou *et* *al,* 2015; Gehart & Clevers, 2019). In both systems, ECs are the most abundant epithelial cell type and play important roles in nutrient absorption and immune homeostasis (Peterson & Artis, 2014).

The intestinal epithelium of both species can be repaired after injury. Studies in *Drosophila* suggest that differentiated ECs represent a source signal to direct ISC division for tissue turnover (Jiang *et* *al,* 2009; Liang *et* *al,* 2017). A recent study revealed that p38 signalling is activated in ECs to integrate multiple types of stress via a Nox‐Ask1‐MKK3‐p38 axis. This EC‐dependent response mediates ISC proliferation and intestinal regeneration (Patel *et* *al,* 2019). Moreover, the Hippo pathway has been identified to play a central role during intestinal regeneration in *Drosophila* and mice (Hong *et* *al,* 2016). In the mouse intestine, Yap, the transcriptional regulator of the Hippo pathway, is dispensable for intestinal homeostasis but required for the regeneration of the Lgr5^+^ ISC pool after damage (Gregorieff *et* *al,* 2015). In a recent work, single‐cell analysis after irradiation revealed a quiescent intestinal cell type, which is activated after injury in a Yap‐dependent manner. This cell type was termed revival stem cell and was shown to be required to replenish the ISC pool and restoration of intestinal function (Ayyaz *et* *al,* 2019). Besides the Hippo pathway, several other signalling pathways are involved in intestinal regeneration (Beumer & Clevers, 2016), such as Wnt signalling. Here, a conserved mechanism between *Drosophila* and mice was identified by Perea *et* *al* (2017), who showed that the Ret receptor tyrosine kinase is required for stem cell proliferation by regulating Wnt signalling. Accordingly, the Ras effector molecules RAL GTPases have also been shown to play a conserved role in promoting Wnt activity, which is important for ISC maintenance and regeneration (Johansson *et* *al,* 2019). These fundamental overlaps in cell type function and actively involved signalling pathways during intestinal homeostasis and regeneration highlight the importance of using both, *Drosophila* and mouse models, to understand intestinal physiology and to guide the development of new clinical approaches.

## Intestinal functions in *Drosophila*

### The anatomy of the *Drosophila* intestine

The adult *Drosophila* intestine is divided into three distinct regions: the foregut, midgut and hindgut. The midgut represents the endodermal counterpart of the adult mouse intestine and is the main focus of this review. The midgut is organized as a tube, composed of an epithelial monolayer, and is separated from the intestinal lumen by a peritrophic matrix, composed of chitin polymers and glycoproteins (Lehane, 1997) to protect itself from bacteria present in the lumen. The intestine is surrounded by a basement membrane and visceral muscles (VMs) (Miguel‐Aliaga *et* *al,* 2018) (Fig 2A). The midgut consists of four main cell types, namely SCs, postmitotic immature enteroblasts (EBs), which differentiate into functional absorptive enterocytes (ECs), and secretory enteroendocrine cells (EECs) (Micchelli & Perrimon, 2006; Ohlstein & Spradling, 2006; Jiang & Edgar, 2012) (Fig 2A). ISCs adhere via integrins to the VM, which, together with EBs and ECs, provide niche factors to the ISCs (Lin *et* *al,* 2008; Cordero *et* *al,* 2012). The merits of the *Drosophila* model for intestinal research are its short life cycle, evolutionarily conserved signalling pathways with human and mice (Neves *et* *al,* 2015), a low level of genetic redundancy and powerful genetic tools to manipulate gene function, using inducible and cell type‐specific approaches.

### Intestinal homeostasis and metabolism

Since ISCs were identified in *Drosophila*, the intestine has been intensively used as a model system to investigate the interplay between metabolism, immune activity and regenerative responses during homeostasis (Micchelli & Perrimon, 2006; Ohlstein & Spradling, 2006). In response to metabolism and nutrient availability, adult *Drosophila* restricts intestinal growth during food scarcity in order to survive in a rapidly changing environment. Additionally, flies have developed an adaptive mechanism to direct symmetric division of stem cells to accelerate intestinal growth when food is abundant (O'Brien *et* *al,* 2011). Furthermore, studies have uncovered a role for the fragile X mental retardation protein/Lin‐28/insulin receptor (InR) axis in sensing nutrients and inducing stem cell expansion, symmetric division and intestinal proliferation (Chen *et* *al,* 2015; Luhur & Sokol, 2015; Luhur *et* *al,* 2017).

**Figure 3:**
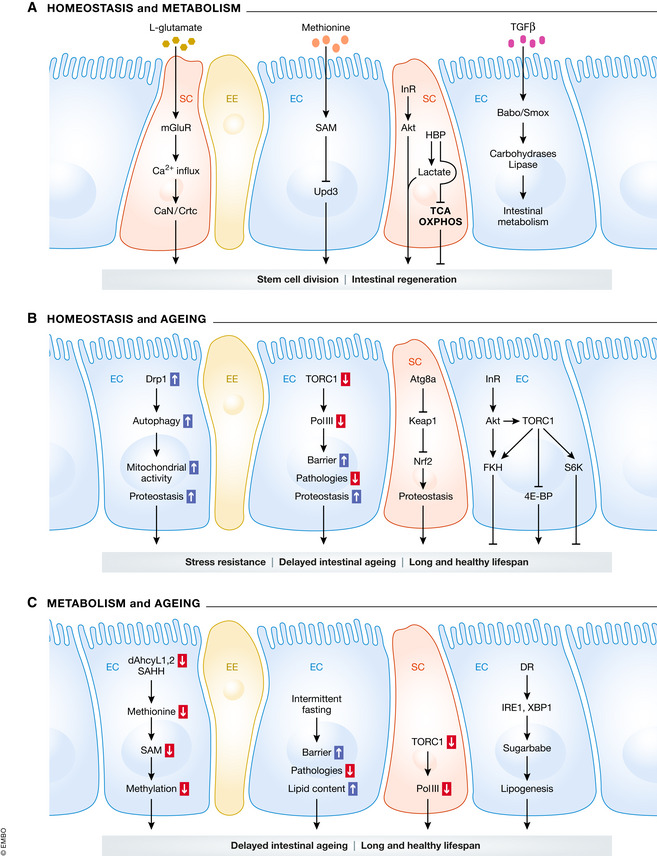
The interplay between metabolism, homeostasis and ageing in Drosophila intestine (A) Intestinal local signals control stem cell division and metabolism to maintain tissue homeostasis. Specifically, metabolic and signalling influxes influence downstream pathways in various cell types to regulate stem cell activity. (B) The pathways involved in autophagy, growth and metabolism improve intestinal function during ageing and extends animal lifespan. (C) The effects of diet restriction on intestinal barrier function and ageing. Blue arrows indicate an upregulation, and red arrows indicate a downregulation.

The role of dietary inputs in the regulation of ISC activity and intestinal epithelial turnover has recently started to be explored. For example, *S*‐adenosyl‐methionine (SAM) from dietary methionine acts as a critical molecule to regulate stem cell division in the midgut by controlling protein synthesis via a unique diphthamide modification on eukaryotic elongation factor 2 (Obata *et* *al,* 2018). SAM also regulates the expression of the JAK/STAT ligand Unpaired 3 (*Upd3*) in ECs, which is known to induce stem cell proliferation upon refeeding (Fig 3A) (Obata *et* *al,* 2018). Moreover, the abundance of food triggers SCs to employ the hexosamine biosynthesis pathway (HBP). Elevated HBP levels then induce a Warburg effect‐like metabolic switch and InR signalling activity to enhance ISC division and intestinal growth (Mattila *et* *al,* 2018) (Fig 3A). Another example of dynamic stem cell response to available metabolites/nutrients is dietary L‐glutamate, which activates the metabotropic glutamate receptor and causes high cytosolic Ca^2+^ to induce SC division and intestinal growth by regulating calcineurin (CaN) and CREB‐related transcriptional co‐activator (Crtc) (Deng *et* *al,* 2015) (Fig 3A). These data highlight the intestine as a dynamic tissue that adjusts SC activity for organ growth in response to food availability.

Intestinal cell populations communicate with each other to cooperatively support tissue regeneration and to regulate metabolic homeostasis (Miguel‐Aliaga *et* *al,* 2018). For instance, the muscle‐derived insulin‐like 3 peptide (dIlp3) activates InR signalling in stem cells, which drives diet‐stimulated intestinal growth (O'Brien *et* *al,* 2011). However, this intestinal SC–muscle interaction is severely impaired upon loss of tachykinin (Tk)‐ or Tk‐producing EECs, suggesting an important role of EECs in diet‐stimulated stem cell division and tissue growth (Amcheslavsky *et* *al,* 2014; Scopelliti *et* *al,* 2019). Indeed, EECs are required for nutrient sensing, and a subset of Dh31‐ and Tk‐expressing EECs are activated in the presence of proteins and amino acids (Park *et* *al,* 2016). These observations highlight the importance of SC‐EECs and SC‐muscle crosstalk in nutrient sensing, metabolic adaptation and organ growth. In addition to these mentioned examples, the communication between intestinal cells may influence other aspects of intestinal homeostasis, such as ageing, immunity and microbiota, which requires further investigation.

Enteroendocrine cells are known to produce hormones in response to external stimuli, such as nutrients, and to regulate local and systemic metabolism. Studies uncovered that EECs control intestinal and systemic lipid homeostasis, eventually affecting animals’ food intake, locomotor activity and lifespan (Song *et* *al,* 2014; Kohyama‐Koganeya *et* *al,* 2015, 2017; Takeda *et* *al,* 2018). In addition, dietary lipids modulate Delta ligand and Notch extracellular domain stability to guide EEC differentiation (Obniski *et* *al,* 2018). Interestingly, ISCs rely primarily on lipid metabolism, more specifically lipolysis, to serve as energy for their maintenance and survival (Singh *et* *al,* 2016). These data demonstrate an important role for EEC in linking dietary lipid or lipid metabolism with stem cell activity and intestinal growth (Amcheslavsky *et* *al,* 2014; Singh *et* *al,* 2016; Obniski *et* *al,* 2018).

In addition to lipid metabolism, other nutrients can influence intestinal growth. For example, yeast ingestion triggers the destabilization of the Misshapen and Warts proteins to allow Yorkie‐dependent intestinal growth (Li *et* *al,* 2018). Also, dietary sugar stimulates transforming growth factor beta (TGFβ) signalling in the intestine to regulate local and systemic metabolism (Chng *et* *al,* 2014; Song *et* *al,* 2017; Song *et* *al,* 2017) (Fig 3A). It was reported that pyruvate metabolism in enterocytes regulates stem cell proliferation by a canonical non‐autonomous activation of JAK/STAT signalling (Wisidagama & Thummel, 2019). A more recent study demonstrates Hodor as a metal sensor in the intestine that controls dietary zinc preference and impacts on food intake and insulin signalling for systemic growth (Redhai *et* *al,* 2020). These findings indicate that dietary nutrients (proteins, lipids and sugars) influence developmental signalling pathways to control intestinal metabolism and growth. Importantly, the majority of these effects influence the ISCs indirectly and rather mediated via other intestinal cell types.

### Intestinal homeostasis and ageing

Ageing is an extremely complex and multifactorial process characterized by a time‐dependent functional decline. Increasing evidence indicates that intestinal regenerative capacity has a profound impact on animal lifespan (López‐Otín *et* *al,* 2013). In *Drosophila*, for instance, a mild reduction in ISC proliferation is associated with extended lifespan; however, strong inhibition of ISC proliferation disrupts intestinal regeneration and reduces lifespan (Biteau *et* *al,* 2010). On a mechanistic level, a study showed that the activation of c‐Jun N‐terminal kinase (JNK) stress response signalling caused homeostatic imbalance and intestinal dysplasia in aged intestines by accumulation of inappropriately differentiated stem cell daughter cells (Biteau *et* *al,* 2008). Increasing evidence indicates that improving mitochondrial biogenesis and proteostasis and limiting endoplasmic reticulum (ER) stress in ISCs can delay stem cell ageing (Rera *et* *al,* 2011; Wang *et* *al,* 2014, 2015; Rodriguez‐Fernandez *et* *al,* 2019). For example, overexpression of *Drosophila* peroxisome proliferator‐activated receptor gamma co‐activator 1 (PGC‐1) homologue (spargel) in ISCs increases mitochondrial activity, delays ageing‐related intestinal barrier dysfunction and extends lifespan in flies. Additionally, somatic SCs coordinate cell cycle arrest with protein clearance to prevent proteostasis failure in ageing flies (Rodriguez‐Fernandez *et* *al,* 2019) (Fig 3B).

In the aged intestine, homologous recombination during mitosis causes loss of heterozygosity in SCs, in turn leading to genetic mosaicism. Likewise, age‐related somatic deletion of DNA sequences and large structural rearrangement often cause gene inactivation (Siudeja *et* *al,* 2015). These genomic modifications of tumour suppressor genes, such as Notch, in SCs can result in tumour growth (Siudeja *et* *al,* 2015). Additionally, the age‐related heterochromatin instability in ECs induces genomic stress, JNK signalling and EC cell death, eventually causing non‐autonomous intestinal stem cell ageing (Jeon *et* *al,* 2018). Therefore, a range of cellular intrinsic cues, including DNA rearrangements and genomic instability, influences ISC ageing. Interestingly, during the functional loss of stem cells in ageing flies, ECs can dedifferentiate into ISCs through amitosis with tissue demand (Lucchetta & Ohlstein, 2017), indicating that the intestine utilizes amitosis as a mechanism to regenerate SCs to restore homeostasis. In addition, the argonaute protein family member Piwi is critical for ensuring heterochromatin maintenance, to suppress retrotransposon activation and prevent DNA damage. Loss of Piwi causes reduced SC activity and loss of their progenies by apoptosis, indicating that the genome integrity is essential for the maintenance and function of aged SCs (Sousa‐Victor *et* *al,* 2017). The same group also reported that restoring JNK/Wdr62/Kif1 governed mitotic spindle orientation in ISCs improves ageing‐related intestinal barrier function and extends lifespan (Hu & Jasper, 2019). Moreover, Filer *et* *al,* described an important link between target of rapamycin kinase complex 1 (TORC1) and RNA polymerase Pol III during ageing. Reducing Pol III activity specifically in ISCs extends the lifespan of flies by reducing aged‐related gut dysfunction (Filer *et* *al,* 2017) (Fig 3B). Collectively, improved mitochondrial activity, restored spindle orientation, reduced stress signalling, limited ER stresses, decreased TORC1/Pol III activity, preserved proteostasis and genomic stability in stem cells can promote intestinal homeostasis, reduce epithelial barrier dysfunction and extend animal lifespan (Biteau *et* *al,* 2008; Rera *et* *al,* 2011; Wang *et* *al,* 2014, 2015; Filer *et* *al,* 2017; Hu & Jasper, 2019; Rodriguez‐Fernandez *et* *al,* 2019).

The autophagy system also plays an important role in ageing (Escobar *et* *al,* 2019). Autophagy is a conserved mechanism that sequesters components of the cytoplasm into autophagosomes and further delivers them to lysosomes for degradation and macromolecule recycling. Autophagy plays important roles during development and disease by catabolizing intracellular components to maintain energy homeostasis (Hansen *et* *al,* 2018). In the context of ageing, intestinal‐specific AMP‐activated protein kinase (AMPK)/autophagy‐related gene 1 (Atg1) activation leads to reduced insulin‐like peptide levels in the brain and increased autophagy in both, the brain and intestine, slows systemic ageing and extends animal lifespan (Ulgherait *et* *al,* 2014). Moreover, the specific TOR inhibitor, rapamycin, induces autophagy in the intestine, reducing SC activity and delaying intestinal ageing in *Drosophila* (Fan *et* *al,* 2015). ISCs employ autophagy to eliminate the accumulation of cells with DNA damage and cell cycle arrest during ageing (Garschall *et* *al,* 2017; Nagy *et* *al,* 2018) (Fig 3B). ISCs with reduced lipolysis initiate JNK‐dependent autophagy and recycling by nearby differentiated cells (Singh *et* *al,* 2016). Recent studies revealed that impaired autophagy‐related pathway results in DNA damage, mitochondrial dysfunction (Rana *et* *al,* 2017) (Fig 3B), activation of mitogen‐activated protein kinase (MAPK) signalling and intestinal hyperplasia (Zhang *et* *al,* 2019) as well as stem cell misdifferentiation and short lifespan (Guo *et* *al,* 2019). These studies suggest that autophagy contributes to DNA damage clearance, recycling of unfit cells, restricting stem cell activity, slowing down tissue ageing and animal lifespan extension.

Inducing mitophagy, the selective clearance of mitochondria by autophagy, has been proposed to also have beneficial effects on organismal fitness, for instance, by improving mitochondrial unfolded protein response and proteolytic homeostasis (Borch *et* *al,* 2017), preventing mitochondrial damage (Koehler *et* *al,* 2017) and mitochondrial DNA mutations as well as enhancing stem cell activity (Hansen *et* *al,* 2018; Kauppila *et* *al,* 2018). NAD^+^ level has been shown to be decreased in the flies with pre‐ageing conditions, while supplementation of NAD^+^ precursors restores mitophagy, stem cell activity and extends fly lifespan, suggesting that metabolic intervention prevents stem cell exhaustion or ageing, which can benefit systemic ageing (Fang *et* *al,* 2019). Autophagy seems to play an essential role in ageing by eliminating unfit cells and improving mitochondrial activity and protein turnover, thereby preventing stem cell and systemic ageing. However, the underlying mechanisms are not completely understood. Given these findings, autophagy plays complex roles in maintaining intestinal homeostasis and ageing, and further detailed investigations are needed to address how the multistep autophagy processes can influence ageing.

### Intestinal metabolism and ageing

Metabolic changes that occur during ageing include mitochondrial function, insulin sensitivity, metabolite shuttling and utilization and are influenced by genetic and environmental variation (Finkel, 2015). In several model organisms, including worms, flies and mice, calorie restriction (CR) has been shown to promote longevity (Tatar *et* *al,* 2014; Finkel, 2015; Kapahi *et* *al,* 2017). In *Drosophila*, dietary restriction induces a metabolic switch towards increased triglyceride usage and protects against age‐related pathologies, leading to lifespan extension (Mair *et* *al,* 2003; Piper & Bartke, 2008). Mechanistically, IRE1/XBP1 ER stress signalling and its downstream effector sugarbabe (Sug) are involved in triglyceride synthesis and their accumulation in ECs (Fig 3C). This mechanism mediates tissue metabolic adaptation that allows for dietary restriction‐induced lifespan extension (Luis *et* *al,* 2016) (Fig 3C). In response to dietary restriction of amino acids, the amino acid deprivation‐activated kinases GCN2 and ATF4 transcriptionally regulate the translational inhibitor 4E‐BP to mediate lifespan extension (Kang *et* *al,* 2017). In addition, intermittent fasting in early life has been shown to increase the lifespan and healthspan of flies with increased resistance to starvation, oxidative and xenobiotic stress and improved age‐associated gut pathologies and barrier function, which are considered as anti‐ageing phenotypes (Catterson *et* *al,* 2018) (Fig 3C). Dietary restriction also leads to dMyc upregulation with enhanced enterocyte cellular fitness and improved intestinal barrier function (Akagi *et* *al,* 2018). Notably, this enhanced enterocyte cellular fitness was sufficient to prevent intestinal ageing and increase the life expectancy of flies (Akagi *et* *al,* 2018). The intestine of female flies maintains more highly active SCs and is more resistant to external challenges than that of male flies, but also shows increased defects during ageing. Moreover, studies have shown that dietary restriction also prevents ageing‐related female gut pathologies and improves the healthspan of females more than it does those of male flies (Regan *et* *al,* 2016; Catterson *et* *al,* 2018). Taken together, dietary restriction induces transcriptional and post‐transcriptional changes on genes involved in metabolic adaptation, for example, facilitating lipogenesis and lipid usage that delays age onset intestinal pathologies and increases organismal lifespan.

Methionine is an essential amino acid in animals, and methionine restriction extends the lifespan of multiple model organisms such as *Caenorhabditis elegans*, rat, mouse and *Drosophila* (Orentreich *et* *al,* 1993; Sun *et* *al,* 2009; Cabreiro *et* *al,* 2013; Lee *et* *al,* 2014; Obata & Miura, 2015; Bárcena *et* *al,* 2018; Parkhitko *et* *al,* 2019). In addition, methionine serves as the precursor for the classical methyl donor SAM for methyltransferase activity, which is converted to *S*‐adenosyl‐homocysteine (SAH) (Obata & Miura, 2015; Parkhitko *et* *al,* 2016; Zhang, 2018). The alteration in SAM levels causes changes in epigenetic regulation, lipid metabolism and protein function (Lu & Mato, 2012). Interestingly, Obata & Miura (2015) reported that the systemic level of SAM is increased in aged flies. Overexpression of glycine *N*‐methyltransferase (Gnmt) enhances SAM catabolism, which suppresses the age‐dependent upregulation of SAM levels, resulting in lifespan extension (Obata & Miura, 2015). In addition, dietary restriction and reduced insulin signalling impede the age‐related SAM increase (Obata & Miura, 2015). Another study demonstrated that methionine metabolism changes dramatically during ageing and that SAH levels are increased in aged flies. An *in vivo* RNAi screen targeting methionine pathway components revealed that the *Drosophila* homologues SAH hydrolase Ahcy, dAhcyL1 and dAhcyL2 are involved in the regulation of lifespan (Parkhitko *et* *al,* 2016) (Fig 3C). Reprogramming of methionine metabolism by inhibiting these SAH hydrolases in *Drosophila* or specifically in the brain and intestine suppresses age‐related SAH increases and leads to lifespan extension (Parkhitko *et* *al,* 2016). In summary, increased SAM/SAH levels are associated with animal lifespan, and the regulation of SAM/SAH levels may be an effective strategy for lifespan extension.

In the context of amino acids, a low supply of optimized protein has been found to increase early life fitness (Piper *et* *al,* 2017). This enhanced dietary amino acid quality is beneficial for animal growth and reproduction without comprising lifespan (Piper *et* *al,* 2017). A systematic transcriptional analysis of *Drosophila* tissues identified conserved GATA family transcription factors that regulate the effect of dietary amino acids on lifespan in response to dietary restriction (Dobson *et* *al,* 2018). Interestingly, restriction of branched‐chain amino acids (BCAAs) also results in lipid accumulation, stress resistance and improved age‐associated gut pathology with extended lifespan (Juricic *et* *al,* 2020). These results demonstrate the importance of limiting dietary amino acid supply for lifespan extension, but the molecular details are still lacking.

In addition to amino acid availability and functionality, insulin activity also plays a central role in metabolism during ageing. In general, lowered insulin activity often results in long‐lived animals (Tain *et* *al,* 2017). Studies also showed that fat body‐specific reduction in protein translation and induction of proteasomal activity in the intestine contribute to the extended lifespan in insulin mutant flies (Essers *et* *al,* 2016; Tain *et* *al,* 2017). Accordingly, overexpression of the proteasome β5 subunit increases proteasome‐related chymotrypsin‐like activity, reduces ubiquitinated protein aggregates and increases the lifespan of adult flies (Nguyen *et* *al,* 2019). Intestinal‐specific upregulation of the FoxA transcription factor homologue fork head (FKH) improves gut barrier function in aged flies and extends lifespan (Bolukbasi *et* *al,* 2017). Mechanistically, FKH is phosphorylated by Akt and TOR, and phosphorylated FKH is required for rapamycin and the reduced insulin signalling‐related anti‐ageing effects (Bolukbasi *et* *al,* 2017) (Fig 3B).

## Intestinal functions in mice

### The anatomy of the mouse intestine

The intestine of the mouse, as in humans, can be separated into the small and large intestine, which includes the colon. In this review, we focus on the small intestine as it has been the prime focus of studies and is the functional equivalent to the *Drosophila* midgut. In contrast to the *Drosophila* midgut, the epithelial monolayer of the small intestine in mice is organized in crypt–villus units (Fig 2B). The villi are finger‐like protrusions that reach into the intestinal lumen to increase the absorptive surface, while crypts (also called crypts of Lieberkühn) are deep invaginations that house the ISCs (Bjerknes & Cheng, 1999; Barker *et* *al,* 2007) at their base (Fig 2B). These units are covered by secreted mucus towards the lumen and basally underlined by visceral muscle and mesenchyme (Fig 2B). Along the crypt–villus axis, seven different cell types can be identified (van der Flier & Clevers, 2009): the crypt base harbours the ISCs, intermingled with Paneth cells, which provide niche signals as well as bactericidal products. As cells are pushed from the stem cell niche and ascend the so‐called transit amplifying (TA) zone, they proliferate and differentiate. Differentiated cell types include secretory cells of the intestine, such as GCs and EECs, which secrete mucus and hormones, respectively, and can be found along the entire crypt–villus axis. Absorptive ECs display the most abundant cell type and are located along the villi. Tuft cells and microfold (M) cells are comparatively rare cell types in the small intestine and are involved in the immune response. Advantages of the mouse as a model for intestinal research are the high physiological and genetic similarity to humans (Nguyen *et* *al,* 2015), strong cell type conservation (Neves *et* *al,* 2015) and the possibility to generate intestinal organoids, which are long‐term 3‐dimensional cell cultures (Sato *et* *al,* 2009) (Fig 2B). Intestinal organoids markedly facilitate the experimental accessibility of the intestinal epithelium and stem cell niche and allow for versatile genetic engineering (Koo *et* *al,* 2012). The generation of organoids displays a stem cell‐driven process that reflects ISC function *in vivo* (Sato *et* *al,* 2009) and allows direct assessment of ISC regenerative capacity by measuring the organoid formation efficiency from a single stem cell or crypts in culture.

### Intestinal homeostasis and metabolism

Over the last years, emerging data have shown that in addition to the well‐known regulatory signalling pathways, such as Notch and Wnt (Vermeulen & Snippert, 2014), energy metabolism likewise contributes to ISC function and epithelial homeostasis. In this context, the intestinal epithelium is influenced by intrinsic cellular metabolic state as well as by extrinsic cues, such as metabolites and nutritional states. ISCs have been proposed to integrate the metabolic status of an organism and respond dynamically to changes in the availability of metabolites and dietary status, such as CR and fasting (Yilmaz *et* *al,* 2012; Igarashi & Guarente, 2016; Mihaylova *et* *al,* 2018), ketogenic and glucose‐supplemented diet (Cheng *et* *al,* 2019), high‐fat diet (Beyaz *et* *al,* 2016) or cholesterol‐rich diet (Wang *et* *al,* 2018).

**Figure 4:**
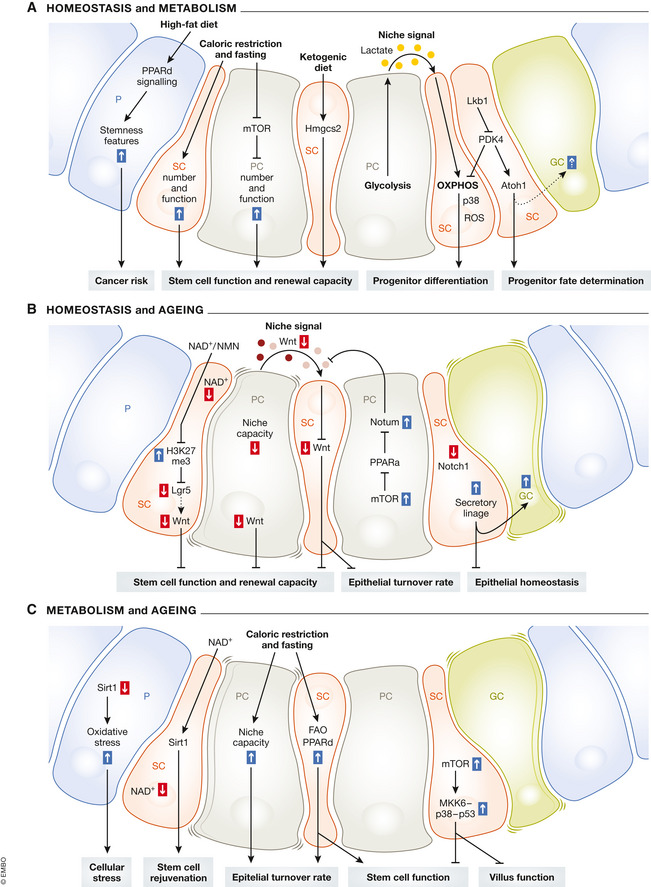
The interplay between metabolism, homeostasis and ageing in the mouse intestine (A) Extrinsic metabolic changes, such as dietary interventions, and intrinsic metabolic states influence the epithelial homeostasis of the intestine. The response of the epithelium to metabolic changes includes stem cell function and renewal capacity, progenitor differentiation and fate determination as well as increased cancer risk. (B) Effects of ageing on the epithelial homeostasis in the mouse intestine include diminished stem cell function and renewal capacity as well as impaired epithelial turnover rate and homeostasis. (C) The effects of metabolites and metabolic changes on ageing‐related phenotypes include induced cellular stress, stem cell rejuvenation, improved epithelial turnover rate and stem cell function as well as impaired villus and stem cell function. Blue arrows indicate an upregulation, red arrows indicate a downregulation, dashed lines indicate suggested biological function, and solid lines indicate shown biological function.

In addition to responding to the nutritional status, ISCs and Paneth cells themselves exhibit different metabolic states, which can drive proliferation and differentiation of intestinal cells (Rodríguez‐Colman *et* *al,* 2017; Schell *et* *al,* 2017). ISCs show high mitochondrial activity (OXPHOS) compared to Paneth cells, which are in turn characterized by pronounced glycolysis (Rodríguez‐Colman *et* *al,* 2017) (Fig 4A). These metabolic profiles directly influence ISC function, as inhibition of mitochondrial activity in ISCs as well as inhibition of glycolysis in Paneth cells leads to diminished organoid formation, which has been used as an assay for ISC function. Moreover, the metabolic compartmentalization of OXPHOS and glycolysis in ISCs and Paneth cells, respectively, appears to be an inherent feature of the stem cell niche. Paneth cells produce high levels of lactate during glycolysis, which they provide to ISCs to fuel their OXPHOS (Rodríguez‐Colman *et* *al,* 2017). Therefore, lactate serves as another niche signal secreted by the PCs, such as Wnts (Gregorieff, 2005), which allows Paneth cells to support ISCs in sensing the metabolic state and controlling differentiation versus proliferation in response to metabolic changes (Fig 4A).

Intestinal stem cells rely on mitochondrial activity to regulate their metabolic rate for the cell fate decision between proliferation and differentiation. On a mechanistic level, mitochondrial activation in ISCs drives differentiation into distinct intestinal cell types, a process that involves p38 (Mapk14) activity and mitochondrial ROS signalling (Fig 4A) (Rodríguez‐Colman *et* *al,* 2017). In this context, blocking oxidative pyruvate metabolism in the mitochondria of ISCs by deleting the mitochondrial pyruvate carrier *Mpc1* increases stem cell proliferation. This results in an expansion of the ISC compartment at the expense of cellular differentiation in the intestine of mice as well as *Drosophila* (Schell *et* *al,* 2017). Thus, mitochondrial OXPHOS facilitates differentiation of intestinal stem cells, while its inhibition leads to an increase in the stem cell pool. This metabolic linkage enables the intestinal epithelium to respond dynamically to changes in metabolic states and adjust the balance between stem cell proliferation versus differentiation. Recently, a link between metabolism and cell fate determination was identified. Here, loss of *Lkb1*, a bioenergetic sensor, in ISCs resulted in a boost of secretory cell types (Gao *et* *al,* 2020). This differentiation bias towards the secretory lineage was independent of Notch signalling, which is instructive during homeostasis for the cell fate decision between absorptive and secretory cell types (Fre *et* *al,* 2005; Pellegrinet *et* *al,* 2011). Instead, the authors identified that during homeostasis, LKB1 inhibits *Atoh1*, the master regulator for the secretory lineage (Yang, 2001), via inhibition of the pyruvate dehydrogenase kinase PDK4 (Fig 4A). PDK4 is an inhibitor of pyruvate dehydrogenase and thereby OXPHOS. Upon loss of *Lkb1* in ISCs, OXPHOS is deregulated and *Atoh1* levels are elevated, resulting in an increased progenitor differentiation into secretory cells (Gao *et* *al,* 2020). Thus, the metabolic state of intestinal stem cells is involved in both, the regulation of proliferation versus differentiation and cell fate determination of progenitor cells. Yet, it remains to be elucidated how OXPHOS levels are timely fine‐tuned in stem cells to first direct towards differentiation, which requires high OXPHOS levels, and subsequently for cell fate decisions, which might require different OXPHOS levels. Moreover, it will be interesting to assess how different dietary regimes influence the metabolic state of ISCs and to what extent intrinsic and extrinsic responses to nutrition are linked and feed into each other.

As observed for the metabolic state, the nutrition status of the organism can also have a direct impact on ISCs or an indirect impact mediated via Paneth cells and niche‐induced changes. For example, CR leads to an increase in ISC and Paneth cell numbers, as well as enhanced ISC function (Fig 4A), identified by improved organoid formation (Yilmaz *et* *al,* 2012; Igarashi & Guarente, 2016). This response to CR involves mechanistic target of rapamycin kinase (mTOR) complex 1 (mTORC1) signalling in Paneth cells and induces a shift of equilibrium towards ISC renewal at the expense of differentiation, for example, into absorptive cells in response to reduced food intake (Fig 4A) (Yilmaz *et* *al,* 2012; Igarashi & Guarente, 2016). These findings further support the hypothesis that Paneth cells have a supportive role in sensing nutritional availability and contribute to a dynamic response by modulating ISC function. In summary, ISCs and Paneth cells communicate in response to CR and mTOR signalling is centrally involved in transmitting this response.

In comparison to CR, short‐term fasting results in a direct stem cell‐intrinsic response, where they switch to fatty acid metabolism (Mihaylova *et* *al,* 2018). This effect was identified by co‐culturing fasted ISCs with *ad libitum* Paneth cells and vice versa to generate intestinal organoids. Thereby fasting alone improved ISC capacity, indicating that the fasting‐mediated response in ISCs is highly dynamic and independent of Paneth cells, in contrast to the CR‐mediated response (Yilmaz *et* *al,* 2012; Igarashi & Guarente, 2016; Mihaylova *et* *al,* 2018). Mechanistically, this metabolic change is facilitated by the activation of peroxisome proliferator‐activated receptor delta (PPARd) signalling and mediated by CPT1A. Moreover, the positive fasting effect in ISCs was mimicked by turning on fatty acid oxidation (FAO) metabolism via drug‐induced activation of PPARd (Fig 4A), revealing options for future drug treatments (Mihaylova *et* *al,* 2018).

In contrast to caloric reduction, increased caloric intake by a high‐fat diet (HFD) has diverse effects on ISCs and Paneth cells, increasing the number of ISCs while reducing the number of Paneth cells (Beyaz *et* *al,* 2016). Elevated stem cell numbers are in line with another study (Mah *et* *al,* 2014), which shows that HFD leads to a higher rate of stem cell divisions and may thereby contribute to an increased risk of intestinal tumours upon HFD. On a mechanistic level, HFD activates PPARd signalling, which activates a restricted ß‐catenin programme in ISCs as well as non‐ISC (Lgr5‐GFP^low^) progenitor cells (Fig 4A). As a result, ISCs become less dependent on Paneth cells, for example during organoid formation. Likewise, progenitor cells showed stemness features (Fig 4A) and were able to form organoids independently. Thus, HFD induces stem cell divisions and thereby potentiates the number of cells of origin for tumours. Moreover, non‐ISC progenitor cells acquire stemness potential and may also contribute to potential sites of tumour initiation (Beyaz *et* *al,* 2016).

Alongside CR, fasting and high‐fat diet, cholesterol availability was recently shown to affect and modulate ISC function and to promote ISC proliferation (Wang *et* *al,* 2018). Moreover, a ketogenic diet was identified to increase ISC number and function, which was counteracted by a glucose‐supplemented diet (Cheng *et* *al,* 2019). Mechanistically, ISCs express high levels of *Hmgcs2*, an enzyme that produces ketone bodies, including beta‐hydroxybutyrate (ßOHB), which in turn inhibits histone deacetylases to sustain Notch signalling and mediate intestinal homeostasis (Cheng *et* *al,* 2019) (Fig 4A). In summary, these studies demonstrate that diet and metabolism have a direct impact on ISC function and influence intestinal homeostasis.

Moreover, the regenerative capacity of the intestinal epithelium can be influenced by CR. Hereby CR‐mediated inhibition of mTORC1 preserves the activity of reserve stem cells, which allows for an improved epithelial regeneration after injury (Yousefi *et* *al,* 2018). Accordingly, genetic *mTOR* disruption severely affects the regeneration capacity after irradiation (Sampson *et* *al,* 2016).

### Intestinal homeostasis and ageing

The effect of ageing in the intestinal epithelium was initially studied by descriptive analyses, which identified changes in the intestinal architecture, such as altered villi structure and cell type composition. Moreover, these age‐related changes were linked to deteriorated ISC activity (Martin *et* *al,* 1998), indicating the importance of ISC function for tissue homeostasis. On a functional level, exhaustion of aged ISCs is displayed *in vivo* as measured by a reduced tissue self‐repair capacity after damage and a slower turnover of the epithelium in aged mice (Nalapareddy *et* *al,* 2017). Moreover, aged ISCs have limited capacity to form organoid cultures *in vitro* (Moorefield *et* *al,* 2017; Nalapareddy *et* *al,* 2017) as well as attenuated differentiation potential estimated by crypt number per organoid (Nalapareddy *et* *al,* 2017; Cui *et* *al,* 2019; Pentinmikko *et* *al,* 2019). On a mechanistic level, stem cell exhaustion can be caused by increased DNA damage. ISCs acquire a high replicative age due the fast renewal rate of the epithelium, which is further potentiated by a reduced DNA damage response of aged ISCs (Watanabe *et* *al,* 2019).

In terms of changes in stem cell number upon ageing, various studies have identified either an ISC expansion and hyperproliferation (Moorefield *et* *al,* 2017), unchanged ISC numbers (Kozar *et* *al,* 2013; Nalapareddy *et* *al,* 2017) or a reduction (Mihaylova *et* *al,* 2018; Pentinmikko *et* *al,* 2019). One reason for these contradictory observations might be due to the different reporter lines used in these studies such as EGFP‐Sox9 (Moorefield *et* *al,* 2017) and Lgr5‐EGFP‐IRES‐CreERT2 (Nalapareddy *et* *al,* 2017; Mihaylova *et* *al,* 2018), as well as the continuous‐labelling approach used by one study (Kozar *et* *al,* 2013). Thus, further investigations are needed to explore the mechanism of ISC exhaustion. The use of currently emerging single‐cell techniques may be one way to address these questions.

The characterization of epithelial homeostasis in aged intestines revealed an increase in Paneth cells (Nalapareddy *et* *al,* 2017; Mihaylova *et* *al,* 2018; Pentinmikko *et* *al,* 2019) and goblet cells (Moorefield *et* *al,* 2017; Nalapareddy *et* *al,* 2017; Igarashi *et* *al,* 2019), which indicates a change in the differentiation potential of ISCs upon ageing and a bias towards the secretory lineage (Fig 4B). This finding is consistent with reduced *Notch1* expression in ISCs (Nalapareddy *et* *al,* 2017), which drives differentiation towards the secretory lineage (Pellegrinet *et* *al,* 2011; VanDussen *et* *al,* 2012). However, it still remains to be elucidated if these changes in ISC cell fate determination and epithelial homeostasis are based solely on ISC‐intrinsic changes or if the surrounding aged stem cell niche is likewise involved.

To identify the molecular mechanism that underlies age‐dependent deterioration of the intestinal epithelium, transcriptomic analyses of fluorescent‐activated cell sorting (FACS)‐sorted ISCs, Paneth and progenitor cells were performed during the last few years (Moorefield *et* *al,* 2017; Nalapareddy *et* *al,* 2017; Kazakevych *et* *al,* 2019; Pentinmikko *et* *al,* 2019). These transcriptomic data revealed that canonical Wnt signalling, in particular *Wnt3*, is reduced in ISCs, Paneth cells and the underlying mesenchyme upon ageing (Fig 4B). Accordingly, WNT3A treatment of organoids from aged mice restored the ageing phenotype of reduced organoid formation efficiency (Nalapareddy *et* *al,* 2017). Mechanistically, aged Paneth cells produce increased levels of *Notum*, a Wnt antagonist, which inhibits canonical Wnt signalling in ISCs (Fig 4B) (Pentinmikko *et* *al,* 2019). The authors further identified that deregulation of *Notum* expression is driven by an altered mTORC1‐PPARa axis in aged Paneth cells. Genetic targeting of *Notum* or exogenous Wnt activation likewise restored ISC function and organoid formation, while genetic activation of mTORC1 specifically in the intestinal epithelium mimicked an ageing phenotype (Pentinmikko *et* *al,* 2019). The reduction in canonical Wnt signalling during ageing might be further enhanced by epigenetic changes since *Lgr5* becomes epigenetically silenced by trimethylation on histone H3K27 (Fig 4B) in intestinal organoids from aged mice (Uchida *et* *al,* 2018).

Interestingly, it was also shown that *Lgr5* expression and ISC function upon ageing could be restored by treatment of organoids with nicotinamide mononucleotide (NMN), a nicotinamide adenine dinucleotide (NAD^+^) intermediate (Uchida *et* *al,* 2018). Accordingly, another recent study showed that NAD^+^ supplementation and activation of the protein deacetylase *Sirt1* improved the functionality of aged ISCs and ameliorated ageing phenotypes (Fig 4B) (Igarashi *et* *al,* 2019). These findings suggest a central role of metabolism during ageing‐associated changes in the intestine and highlight the importance of analysing this connection in the future in more detail.

### Intestinal metabolism and ageing

Metabolism has been shown to have essential effects on ISC function and intestinal epithelial homeostasis, which are likewise altered by ageing. Thus, the interaction of metabolism and ageing appears to present an intriguing crosstalk and might provide intervention points to prevent age‐associated deterioration of the intestinal epithelium, as observed in elderly individuals (Soenen *et* *al,* 2016).

Fasting has been shown to improve ISC function and capacity to form organoids via activation of PPARd/FAO signalling in young mice (Mihaylova *et* *al,* 2018). In aged mice, both fasting and direct activation of the PPARd/FAO axis by a small molecule agonist improved ISC function as measured by the capacity of organoid formation (Fig 4C). However, short‐term fasting of 24 h was less effective in improving ISC function in aged mice. This indicates that the positive effect of fasting on ISCs also occurs in aged animals and ameliorates age‐associated deterioration, but it is less dynamic than that in young animals (Mihaylova *et* *al,* 2018).

Moreover, *in vivo* fate‐mapping experiments in aged fasted and control mice revealed an increased number of labelled progenitor cells after short‐term fasting (Mihaylova *et* *al,* 2018), hinting towards an improved epithelial turnover rate upon reduced caloric uptake (Fig 4C). This observation indicates that fasting has a positive effect on ISC capacity and epithelial homeostasis not only in young mice but also in aged animals and is able to ameliorate the ageing phenotypes in ISCs.

Another mechanism of how fasting, and thereby dampened mTORC1 activity (Yilmaz *et* *al,* 2012), improves ISC function was recently reported (He *et* *al,* 2020). The authors identified that increased mTORC1 signalling in aged ISCs and transit amplifying cells induces the MKK6‐p38‐p53 stress response pathway activity. Enhanced p53 expression caused ISC exhaustion and reduced villi functionality, which is accompanied by reduced nutrient absorption upon ageing (Fig 4C). The ageing phenotypes were partially reversed by rapamycin treatment or p38 inhibition, opening a door for therapeutic intervention beyond CR.

At the molecular level, the NAD‐dependent protein deacetylase *Sirt1* has been shown to mediate the positive effect of NAD^+^ supplementation in aged ISCs towards rejuvenation (Fig 4C) (Igarashi *et* *al,* 2019). Interestingly, compared to control mice, *Sirt1* knockout (KO) mice display elevated numbers of Paneth cells from mid‐age (5–8 months) on (Wellman *et* *al,* 2017), which mimics the differentiation bias towards the secretory lineage observed upon ageing (Nalapareddy *et* *al,* 2017; Mihaylova *et* *al,* 2018; Pentinmikko *et* *al,* 2019). Moreover, upon loss of *Sirt1*, other ageing phenotypes in aged mutant mice compared to those of aged control mice such as the NRF2‐mediated oxidative stress response (Fig 4C) are enhanced (Wellman *et* *al,* 2017). These observations suggest that loss of *Sirt1* mimics ageing‐related phenotypes in young organisms and, during ageing, enhances them. A similar effect of a stronger transcriptional response upon intervention in an aged versus young organism was seen in the context of high‐fat diet. Here, the transcriptional response, in terms of numbers of differentially regulated genes, upon high‐fat diet was stronger in aged mice than in young mice (Steegenga *et* *al,* 2012). These findings suggest that the regulatory network of ISCs becomes less robust and more susceptible to external influences with age, correlating with an overall decline in animal fitness upon ageing. This is in the context of ageing an interesting aspect as changes on the organismal level are intriguing targets for ageing interventions. Thus, it will be important to assess whether this effect is intestine specific or a general organismal mechanism.

### Comparative insights

Knowledge on intestinal homeostasis, ageing, metabolism and the interplay of the latter from *Drosophila* and mouse gives valuable insights in overall invertebrate and vertebrate biology. The two systems share some overlap in regulatory control and physiological responses while also displaying differences in how they respond to metabolic changes and which ageing phenotypes they develop in the intestine.

One central experimental difference between both model systems is currently how ageing phenotypes are assessed. While in *Drosophila* lifespan curves are indicative for an ameliorated ageing phenotype, such a readout is not feasible in a similar throughput in the mouse system. Here, in contrast, stem cell capacity assays are used to indirectly assess if the ageing phenotype, such as reduced stem cell function, is improved upon a respective intervention. This difference is important to consider when comparing studies from both model organisms, but offers likewise a valuable opportunity to address molecular mechanisms in depth by cross‐species investigations. This is further supported by the great potential of genetic engineering in *Drosophila*, which can be achieved in a distinctly shorter time frame and on a broader spectrum (Port *et* *al,* 2020) compared to mice, providing options for complement studies.

Intestinal homeostasis is strongly linked and influenced by metabolic changes in *Drosophila* and mouse. Here, it becomes apparent that ISCs in both systems are partially dependent on other intestinal cell types to sense and respond to changes in nutrient availability. In *Drosophila*, stem cells have an instructive crosstalk with EECs for dynamic adaptation to metabolic changes (Amcheslavsky *et* *al,* 2014), while in mice Paneth cells are involved in this process (Yilmaz *et* *al,* 2012). Besides such extrinsic metabolic regulations by dietary alterations, intestinal stem cells are also controlled by intrinsic metabolic states. In *Drosophila* and mice, deletion of the mitochondrial pyruvate carrier Mpc1, which blocks mitochondrial OXPHOS, triggers a proliferative response and an increase of the stem cell pool (Schell *et* *al,* 2017). Similar effects on stem cell proliferation are observed in *Drosophila* and mice upon CR. In *Drosophila*, this is due to a switch from oxidative to glycolytic metabolism (Mattila *et* *al,* 2018).

Intestinal homeostasis is controlled by the number and proliferation rate of stem cells, not only in response to metabolic changes, but also during ageing. In both systems, mTOR signalling plays a central role during intestinal ageing. In *Drosophila*, manipulation of TORC1 activity directly in the ISCs delays ageing‐related intestinal dysfunction and improves lifespan (Xi *et* *al,* 2019). In comparison, in aged mice, elevated mTORC1 activity in Paneth cells partially accounts for reduced ISC capacity by dampening of canonical Wnt signalling activity in the stem cells. This is caused by an mTORC1‐mediated enhanced secretion of the Wnt antagonist NOTUM (Pentinmikko *et* *al,* 2019), and in addition, Paneth cells express less *Wnt3* as a niche signal for ISCs (Nalapareddy *et* *al,* 2017).

From the above, one can conclude that mouse ISCs are in the context of metabolic as well as ageing‐related changes greatly influenced by Paneth cells and form a strong dependent relationship. While the *Drosophila* intestine is devoid of Paneth cells, stem cells display crosstalks with all the different cell types in distinct contexts. If mouse ISCs are likewise influenced by other intestinal cell types beside Paneth cells during metabolic changes and ageing, remains to be elucidated.

Metabolic changes influence the intestinal epithelium over the entire lifespan of *Drosophila* and mice, and interplay with cellular alterations upon ageing. In *Drosophila* and mice, supplementation of NAD^+^ prevents stem cell ageing, mediated via a Sirt1‐dependent mechanism and probably relying on the regulation of mitophagy (Fang *et* *al,* 2019; Igarashi *et* *al,* 2019). In terms of dietary changes, fasting causes a metabolic switch in both systems with positive effects on lifespan and ageing phenotypes. In *Drosophila,* this intervention increases the usage of triglycerides, leading to lifespan extension. In mice, fasting triggers fatty acid oxidation, which similarly improves the function of aged ISCs, indicated by increased organoid formation efficiency. While in *Drosophila* it is well established that a reduction in Methionine levels in the intestine are associated with improved lifespan (Parkhitko *et* *al,* 2016), in mice it is so far only associated with improved barrier function in young high‐fat‐fed mice (Yang *et* *al,* 2019). However, since reduced intestinal barrier function is an age‐related phenotype, future studies might shine light on a potential correlation of methionine reduction and improved ISC function during ageing.

### Intestinal barrier function in disease and ageing

The maintenance of intestinal barrier function, including its metabolic and immune functions, is essential for organismal health. Intestinal barrier failure has been described as a pathological hallmark of ageing (Chelakkot *et* *al,* 2018). Dysfunction of the intestinal barrier is associated with local and systemic disorders, including inflammatory bowel disease, obesity, metabolic diseases, liver disease, and lung and brain dysfunctions (Nicoletti, 2015; Chelakkot *et* *al,* 2018; Thaiss *et* *al,* 2018). In addition, the age‐related intestinal dysbiosis is associated with intestinal barrier dysfunction in both *Drosophila* and mice (Clark *et* *al,* 2015; Thevaranjan *et* *al,* 2017). However, the mechanisms of age‐related increases in intestinal barrier dysfunction and how the intestinal barrier maintains tissue homeostasis upon injury remain unclear.

### The intestinal epithelial barrier in *Drosophila*

**Figure 5:**
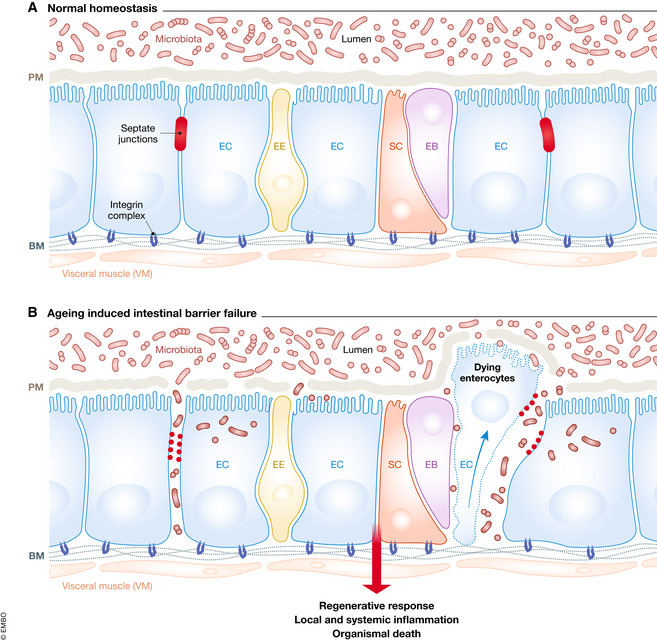
Ageing‐associated intestinal barrier failure (A) Under normal homeostasis, the intact and healthy intestinal barrier maintains epithelial function and prevents bacterial translocation. (B) Ageing causes septate junctions loss, intestinal barrier dysfunction, microbial dysbiosis, systemic infection and animal death.

The intestinal epithelium forms a selectively permeable barrier that consists of mucus and epithelial cells to allow nutrient absorption and prevent infection by pathogens (Nicoletti, 2015). The *Drosophila* intestine also contains the peritrophic matrix (PM), a chitinous layer that protects the tissue from physical damage (Fig 5A) (Buchon *et* *al,* 2013). Intestinal barrier failure leads to immune activation and systemic metabolic defects, including impaired insulin signalling activity, which may link epithelial barrier defects to ageing (Rera *et* *al,* 2012). Furthermore, Clark *et* *al* (2015) reported that alterations in microbial composition occur prior to age‐related intestinal barrier defects, resulting in immune activation and impaired excretory function. This age‐related intestinal barrier defect eventually causes systemic immune activation and animal death (Fig 5B) (Clark *et* *al,* 2015).

To understand the mechanical basis for maintaining the epithelial barrier, several studies have specifically focused on the role of septate junction proteins. For instance, study has demonstrated that barrier integrity relies on the correct positioning of septate junctions in invertebrates and tight junctions in vertebrates (Chen *et* *al,* 2018). Alterations in the levels or localization of junction‐associated proteins can lead to impaired intestinal barrier function, ageing‐related ISC proliferation and misdifferentiation (Izumi *et* *al,* 2012; Yanagihashi *et* *al,* 2012; Byri *et* *al,* 2015; Resnik‐Docampo *et* *al,* 2017, 2018). For example, Bonnay *et* *al* (2013) demonstrated that the septate junction protein big bang (bbg) is required to maintain barrier function. Loss of the septate junction protein Snakeskin (Ssk) leads to rapid intestinal barrier failure, gut morphology changes, microbial dysbiosis and reduced lifespan (Salazar *et* *al,* 2018).

Recent studies have also revealed an important role of the basal lamina (BM) in maintaining intestinal barrier function (Kiss *et* *al,* 2016). The BM is a layer of extracellular matrix secreted by intestinal epithelial cells. Loss of one key component of BM, type IV collagen (col4a1), leads to a shortened gut, impaired intestinal barrier, immune‐related gene activation and reduced lifespan (Kiss *et* *al,* 2016). *Drosophila* transglutaminase (TG) crosslinks the PM protein Drosocrystallin (Dcy) to form the gut mucosal barrier (Kuraishi *et* *al,* 2011; Shibata *et* *al,* 2015). Intestinal‐specific loss of Dcy or TG induces cell death, disrupts the epithelial barrier and causes death of flies upon bacterial challenge (Shibata *et* *al,* 2015). Consistently, loss of the O‐glycosyltransferase PGANT4 in the larval gut impairs the secretion of PM components, thereby leading to intestinal barrier dysfunction (Zhang *et* *al,* 2017). Studies have also shown that JNK activity is required for stimuli‐induced intestinal barrier dysfunction followed by intestinal inflammation and microbial dysbiosis. Reducing JNK activity restores intestinal barrier function and host–microbe homeostasis and extends animal lifespan (Zhou *et* *al,* 2017; Zhou & Boutros, 2020). Hence, these data support an important role of epithelial barrier function in the maintenance of intestinal homeostasis to ensure proper nutrient uptake and organismal health during age and challenge conditions.

### Intestinal barrier function in mice

Intestinal epithelial cells in mice are connected by tight junctions, including zonula occludens (ZOs), occludins, claudins and junctional adhesion molecules (JAMs) (Tran & Greenwood‐Van Meerveld, 2013). These junction proteins form a continuous and complex architecture with the membrane cytoskeleton to maintain intestinal barrier integrity (Günzel & Yu, 2013; Chelakkot *et* *al,* 2018). The tight junction complexes maintain barrier integrity by interactively cooperating with cytoplasmic peripheral membrane proteins and cytoskeleton components such as F‐actin and myosin (Günzel & Yu, 2013). The disruption of tight junctions leads to increased intestinal permeability and the development of intestinal inflammatory diseases (Zeissig *et* *al,* 2007; Mir *et* *al,* 2016; Almousa *et* *al,* 2018; Chelakkot *et* *al,* 2018). For example, mice lacking junctional adhesion molecule A (JAM‐A) showed increased intestinal permeability with enhanced proinflammatory signatures, such as accumulation of CD4^+^ T cells and upregulation of inflammatory cytokines (Khounlotham *et* *al,* 2012). Additionally, mutations in genes related to tight junction complexes cause gut barrier dysfunction and often lead to distant organ failure and critical diseases (Cunningham & Turner, 2012; Su *et* *al,* 2013; Jin & Blikslager, 2016; Yoseph *et* *al,* 2016; Lorentz *et* *al,* 2017). In contrast, the expression of occludin prevents stimuli‐induced defects in intestinal permeability and improves intestinal function (McCarthy *et* *al,* 1996; Marchiando *et* *al,* 2010). These results indicate that reinforcing tight junction function is essential for improving intestinal physiology and may provide a target for the treatment of inflammation, systemic infection and healthspan extension. An age‐related increase in intestinal barrier dysfunction has been described in both animal and human studies (Parrish, 2017). Further investigations have revealed that advanced age promotes not only intestinal barrier failure but also inflammation, microbial translocation and systemic infection in various model organisms, including *Drosophila*, mice and monkeys (Tran & Greenwood‐Van Meerveld, 2013; Clark *et* *al,* 2015; Mitchell *et* *al,* 2017; Wen *et* *al,* 2019). It remains to be assessed whether developmental signalling pathways, including Wnt pathways, are involved in maintaining the intestinal barrier, similar to its role during skin homeostasis (Augustin *et* *al,* 2013).

## Outlook

Maintaining intestinal homeostasis is essential as its deterioration followed by epithelial barrier failure has severe detrimental consequences for the whole organism. Ageing and associated metabolic changes influence the physiological responses of the whole organism, ranging from the behaviour of stem cells to changes in plasticity and environmental responses, thereby impacting on intestinal homeostasis. An important frontier and focus of recent research is the communication between organs to get a better understanding how gut homeostasis is linked to overall organismal health. Moreover, it will be important to understand whether and how well the insight from model organisms can be translated to human intestinal homeostasis and ageing. Metabolic and signalling pathways implicated in intestinal homeostasis are often highly homologous in vertebrates and invertebrates and the high frequency of gene homology between *Drosophila*, mouse and human (Tables 1 and 2) provide a reliable base to identify conserved processes. Studies that assess both invertebrate and vertebrate systems (Perea *et* *al,* 2017; Schell *et* *al,* 2017; Johansson *et* *al,* 2019) are also instrumental in the identification of genes that act in conserved processes during intestinal homeostasis.

The *Drosophila* intestine and its interaction with other organs has been used as a model in recent years to analyse principal mechanisms of inter‐organ communication (Liu & Jin, 2017). For example, it was found that pro‐hormones secreted by EEs are associated with lipid storage and systemic lipid metabolism in the fat body, the functional homolog of the mammalian liver. Loss of tachykinins, which are secreted by EEs in the intestine, results in elevated lipid levels in the fat body, while an increase in tachykinin levels causes a decrease in systemic lipid storage (Amcheslavsky *et* *al,* 2014). The gut–brain axis is another intriguing example of inter‐organ communication. Here, important roles have been attributed to insulin‐like peptides (Dilps), which are mainly derived from insulin‐producing cells in the brain. Dilps have also been found to be involved in the damage‐induced self‐renewal of ISCs (Amcheslavsky *et* *al,* 2009) and are generally associated with organismal health and lifespan, paralleling the positive effect of insulin on longevity (Tain *et* *al,* 2017). A recent study underlined the role of the gut–brain axis in mammals, showing its involvement in lowering hepatic glucose production upon high‐fat diet in rats via inhibition of mTOR signalling in the upper small intestine (Waise *et* *al,* 2019). Further studies of organ‐to‐organ communication will be required to unravel how metabolism and ageing as physiological parameters for organismal health influence intestinal homeostasis and homeostatic regulation in the whole organism.

Recent advances in *ex vivo* culturing of 3‐dimensional organoids (Clevers, 2016) and combining different human tissues as organs‐on‐a‐chip (Takebe *et* *al,* 2017) might open the door for physiologically relevant testing of drugs to influence the metabolism and ageing‐dependent inter‐organ communication.

Another focus of intensive research is the intestinal microbiome. The intestine of animals harbours a complex microbial community that is involved in host food digestion, metabolic adaptation and immune system modulations (Leulier *et* *al,* 2017). Increasing evidence suggests an important role of host–microbe interaction in ageing using various model systems, such as nematode, fly and mouse (Cabreiro *et* *al,* 2013; Gusarov *et* *al,* 2013; Clark *et* *al,* 2015; Nguyen *et* *al,* 2015). These studies proposed that alterations in microbiota (dysbiosis) could represent an important regulator of ageing‐related processes. In addition, recent studies already began to explore effective ways to improve animal longevity through targeted microbiome alterations (Bana & Cabreiro, 2019; Pryor *et* *al,* 2019).

We particularly highlight the role of the intestinal epithelial barrier as an integrator of various internal and external signalling during ageing. Strengthening of this barrier function can be an effective strategy to prevent intestinal inflammation and delay ageing‐related intestinal pathology. Further molecular studies, combining the strengths of genetic model systems and *ex vivo* organoid cultures, can lay the basis for the development of agents that sustain and improve intestinal barrier function and mitigate its decay in disease and ageing.

## Conflict of interest

The authors declare that they have no conflict of interest.

**Glossary**
(m)TORC1: (mechanistic) target of rapamycin complex 1
AL: *ad libitum*
AMPK: AMP‐activated protein kinase
BM: basal lamina
BMP: bone morphogenetic protein
CR: caloric restriction
CREB: cyclic AMP‐responsive element‐binding protein
Dilps: insulin‐like peptides
EB: enteroblast
EC: enterocyte
EEC: enteroendocrine cell
EGF: epidermal growth factor
ER: endoplasmatic reticulum
FAO: fatty acid oxidation
FKH: homologue fork head
GC: goblet cell
HBP: hexosamine biosynthesis pathway
HFD: high‐fat diet
InR: insulin receptor
ISC: intestinal stem cell
JAK/STAT: Janus kinase/signal transducers and activators of transcription
JAM: junctional adhesion molecules
JNK: c‐Jun N‐terminal kinase
MAPK: mitogen‐activated protein kinase
NAD: nicotinamide adenine dinucleotide
NMN: nicotinamide mononucleotide
OXPHOS: oxidative phosphorylation
PC: Paneth cell
PM: peritrophic matrix
PPAR: peroxisome proliferator‐activated receptor
ROS: reactive oxygen species
SAH: *S*‐adenosyl‐homocysteine
SAM: *S*‐adenosyl‐methionine
SC: stem cell
TA: transit amplifying
TGFβ: transforming growth factor beta
TG: transglutaminase
Tk: tachykinin
TOR: target of rapamycin
VM: visceral muscle
Wnt: wingless‐related MMTV integration site 1
ZO: zona occludens

**In need of answers**

1. During metabolic changes, the EECs in *Drosophila* and Paneth cells in mice convert instruction to the ISCs for cellular responses. How do intestinal stromal cells, immune cells and microbes impact on the metabolic state of stem cells to influence their behaviour?
2. In the aged murine intestine, Paneth cells impact on ISCs and account for their reduced capacity; however, it remains to be unravelled what causes the age‐related changes in the Paneth cells. Moreover, it is so far unknown what impact the aged underlying mesenchyme, immune cells and enteric nervous system have on the regeneration potential of the aged intestinal epithelium?
3. In ageing conditions, dynamic metabolic changes must occur in various intestinal cell types, especially stem cells. Taking advantage of single‐cell technologies, functional omics can be applied to these cells not only for investigating the underlying mechanisms in the ageing process but also uncovering better strategies to prevent ageing effects.
4. How do ageing‐related changes in the microenvironment or stem cell niche influence intestinal disorders such as inflammatory bowel diseases or cancer? Also, how is the cross communication between the microenvironment, stem cell niche and stromal cells contributing to disease progression?

## Supporting information



Appendix
