# Supplementary material for: Ageing, metabolism and the intestine
Source: EMBO Rep. 2020 Jun 21;21(7):e50047. doi: 10.15252/embr.202050047 (PMC7332987; doi:10.15252/embr.202050047)
Supplement: Supplementary file 1 — Appendix [file EMBR-21-e50047-s001.pdf]

Table 1:

Drosophila gene	Mouse homolog	Homology score	Human homolog	Homology score
Metabolism				
Ahcy	Gm4737	+++	AHCY	+++
AhcyL1	AhcyL1	+++	AHCYL1	+++
AhcyL2	AhcyL2	+++	AHCYL2	+++
Crtc	Crtc1	+++	CRTC1	+++
mGluR	Grm3	+++	GRM3	+++
CanB	Ppp3r1	+++	PPP3R1	+++
Drp1	Dmn1l	+++	DNM1L	+++
Gcn2	Eif2ak4	+++	EIF2AK4	+++
park	Prkn	+++	PRKN	+++
Pgam5	Pgam5	+++	PGAM5	+++
Pgant4	Galnt10	+++	GALNT10	+++
Pink1	Pink1	+++	PINK1	+++
Signaling				
AMPKα	Prkaa2	+++	PRKAA2	+++
Ilp3	Igf1	+	INS	+
InR	Insr	+++	INSR	+++
Pi3K21B	Pik3r3	+++	PIK3R3	+++
srl	Pprc1	+++	PPRC1	+++
Tor	Mtor	+++	MTOR	+++
Thor	Eif4ebp1	+++	EIF4EBP1	+++
Dl	Dll1	+++	DLL1	+++

N	Notch1	+++	NOTCH1	+++
msn	Tnik	+++	TNIK	+++
wts	Lats2	+++	LATS1	+++
yki	Yap1	+++	YAP1	+++
Upd3	Il-6	N/A	IL-6	N/A
hop	Jak2	+++	JAK2	+++
Stat92E	Stat5b	+++	STAT5B	+++
bsk/JNK	Mapk8	+++	MAPK10	+++
Myc	Myc	++	MYC	++
Other functions				
Brf	Brf1	+++	BRF1	+++
eEF2	Eef2	+++	EEF2	+++
FoxK	Foxk1	+++	FOXK2	+++
Ire1	Ern1	+++	ERN1	+++
Xbp1	Xbp1	+++	XBP1	+++
Atg1	Ulk1	+++	ULK1	+++
bbg	Pdzd2	++	PDZD2	+
Col4a1	Col4a1	+++	COL4A6	+++
crc	ATF4	++	ATF4	++
Piwi	Piwil1	+++	PIWIL3	+++
prom	Prom1	+++	PROM2	+++
Tk	Tac1	+	TAC1	+
Tg	Tgm1	+++	TGM1	+++
Wdr62	Wdr62	+++	WDR62	+++

Table 1: *Drosophila* genes discussed in the review with their predicted homolog in Mouse and Human (Homology Score based on flybase.org algorithm). All genes are linked to their respective NCBI gene entry. Homology score: 1 - 2: +, 3 - 5: ++, 6 - 15: +++

Table 2

Mouse gene	*Drosophila* homolog	Homology Score	Human homolog	Homology Score
Metabolism				
Mtor	Tor	+++	MTOR	+++
Stk11 (Lkb1)	Lkb1	+++	STK11	+++
Mpc1	Mpc1	+++	MPC1	+++
Pdk4	Pdk	+++	PDK4	+++
Cpt1a	whd	+++	CPT1A	+++
Ppard	Eip75B	++	PPARD	+++
Ppara	Eip75B	++	PPARA	+++
Sirt1	Sirt1	+++	SIRT1	+++
Hmgcs2	HMGS	+++	HMGCS2	+++
Signalling				
Wnt3	wg	+	WNT3	+++
Lgr5	rk	+++	LGR5	+++
Ctnnb1	arm	+++	CTNNB1	+++
Notum	Notum	+++	NOTUM	+++
Notch1	N	+++	NOTCH1	+++
Atoh1	cato	+++	ATOH1	+++
Sox9	Sox100B	++	SOX9	+++
Mapk14 (p38)	p38b	+++	MAPK14	+++
Map2k6 (MKK6)	lic	+++	MAP2K6	+++
Trp53	p53	++	TP53	+++

Table 2: Mouse genes discussed in the review with their predicted homolog in *Drosophila* and Human (Homology Score based on flybase.org algorithm). All genes are linked to their respective NCBI gene entry. Homology score: 1 - 2: +, 3 - 5: ++, 6 - 15: +++
